# Study of helminth eggs ( *Ascaris suum)* inactivation by anaerobic digestion and electrochemical treatment

**DOI:** 10.12688/gatesopenres.14573.2

**Published:** 2024-02-12

**Authors:** Prajakta Pratap Patil, Srikanth Mutnuri

**Affiliations:** 1 Faecal Sludge Management Laboratory, Department of Biological Sciences, Birla Institute of Technology & Science Pilani K K Birla Goa Campus, NH17 B, Zuarinagar, Goa, 403726, India

**Keywords:** anaerobic digestion, Ascaris suum, electrochemical cell, faecal sludge, helminth

## Abstract

**Background:**

The use of insufficiently treated wastewater or Faecal sludge in agriculture raises concerns because of the pathogen content. Helminth eggs (HE) are one of the most crucial pathogens for ensuring public health and safety. Widely used disinfection treatment methods do not guarantee the complete inactivation of helminth eggs. The current study evaluated the effectiveness of anaerobic digestion and electrochemical process on helminth ( *Ascaris suum*) egg inactivation.

**Methods:**

Lab-scale biochemical methane potential (BMP) assay was conducted by spiking *A. suum* eggs in a serum bottle. Total solid (TS), volatile solid (VS), pH, biogas production and its composition, and volatile fatty acids (VFA) were analyzed along with *A. suum* inactivation every third day for the initial 15 days and fifth day for 45 days. In the second set of experiments, a hypochlorite (4700 ppm) solution was generated by electrolysis of aqueous NaCl solution in a membrane-less electrochemical cell. The hypochlorite was diluted (940, 470, 235, and 156ppm) in wastewater, spiked with *A. suum* eggs and then examined for inactivation at regular intervals.

**Results:**

The results of the anaerobic digestion treatment documented 98% inactivation of *A. suum* eggs (0.15 eggs/mL) in 35 days and remained at 0.14 eggs/mL until day 45. Correlation analysis revealed a positive relationship between non-viable eggs and pH and a negative relationship with all the other parameters. Electrochemical treatment achieved 10% inactivation at 940 ppm concentration in 24h.

**Conclusions:**

This study revealed that the inactivation of *A. suum* eggs by anaerobic digestion or electrochemical treatment is a combined effect of more than one parameter.

## Introduction

Faecal sludge and wastewater reuse for agriculture, land irrigation, and groundwater recharge are becoming standard practices worldwide. When dealing with treated faecal sludge or wastewater the most crucial parameter to ensure safety is the pathogen content.

Coliform is the main bacterial pathogen routinely monitored in wastewater treatment plants. Another important group of pathogens monitored regularly are parasites (Helminths). Helminthiasis is a neglected tropical disease caused by helminths ( *Ascaris lumbricoides* (roundworm *), Necator americanus* (hookworm), *Ancylostoma duodenale,* and *Trichuris trichiura* (whipworm)) along with others. These worms are responsible for malnutrition, anemia, and impaired cognitive development in humans ( WHO, 2012; WHO, 2015). Helminths have been monitored since WHO published guidelines to control the number of helminth eggs in 2006. It is advised to limit the content to one helminth egg (HE) per litre of water (1 HE/L) and one egg per gram of total dry solids (1 HE/g TS) for sludge when applying wastewater, excreta, or sludge to crops for raw consumption ( WHO, 2006). When comparing different types of helminths and the percentage infecting humans, *Ascaris lumbricoides* is the most commonly found helminth ( Jimenez *et al*., 2002) and it is the most prevalent parasite infection in India ( Salam & Azam, 2017). Several studies have used *Ascaris suum* (which can infect pigs) as the surrogate helminth to check the efficiency of wastewater treatment processes as it resembles the most resistant infectious helminth in humans, *Ascaris lumbricoides* ( Kato *et al*., 2003; Popat *et al*., 2010; Manser *et al*., 2015). After conducting a comprehensive review of the existing literature, we proceeded to conduct experiments in this research using *A. suum*.

Some technologies are available to inactivate helminth eggs. However, there is still a considerable gap, leading to untreated water being discharged into river bodies. The methods for inactivating helminth eggs currently being used are either costly or subpar. Technology advancement is required to ensure the complete inactivation of helminth eggs and to make treatment methods sustainable and affordable.

Anaerobic digestion (AD) is one of the most comprehensive technologies used for the treatment of helminth eggs ( Olsen & Nansen, 1987; Ruiz-Espinoza *et al*., 2012; Machnicka & Grübel, 2022). Despite being the most used technology, AD's effectiveness against helminth eggs has not been extensively studied. Anaerobic conditions, temperature, pH, and intermediate products (volatile fatty acids, ammonia), which form during anaerobic digestion, help in the inactivation of pathogens including helminths ( Pecson & Nelson, 2005; Fidjeland *et al*., 2015; Rojas-Oropeza *et al*., 2017). This research conducted a process of co-digestion, combining faecal sludge with food waste, aiming to enhance the biogas production. This approach was adopted due to concerns that using only faecal sludge might not generate an adequate amount of biogas. Electrochemical (EC) techniques are also being used to treat pathogens in wastewater efficiently ( Lin *et al*., 2020; Fores *et al*., 2023). When anode and cathode electrodes are subjected to a source of current, free radicals (hydroxyl radicals) are produced. These radicals are not specific to recalcitrant organic compounds and cell components, causing pathogen inactivation. The electrochemical process can result in the production of hypochlorite and the change in the pH of the solution together leads to the inactivation of HE ( Talekar *et al*., 2018).

This study aimed to understand the role of anaerobic digestion and electrochemical process in helminth egg ( *A. suum*) inactivation. 

## Methods

### Anaerobic digestion: biochemical methane potential (BMP) assay

**Figure 1.**
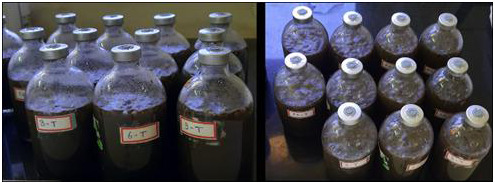
The BMP bottle set up at lab scale

**Figure 2.**
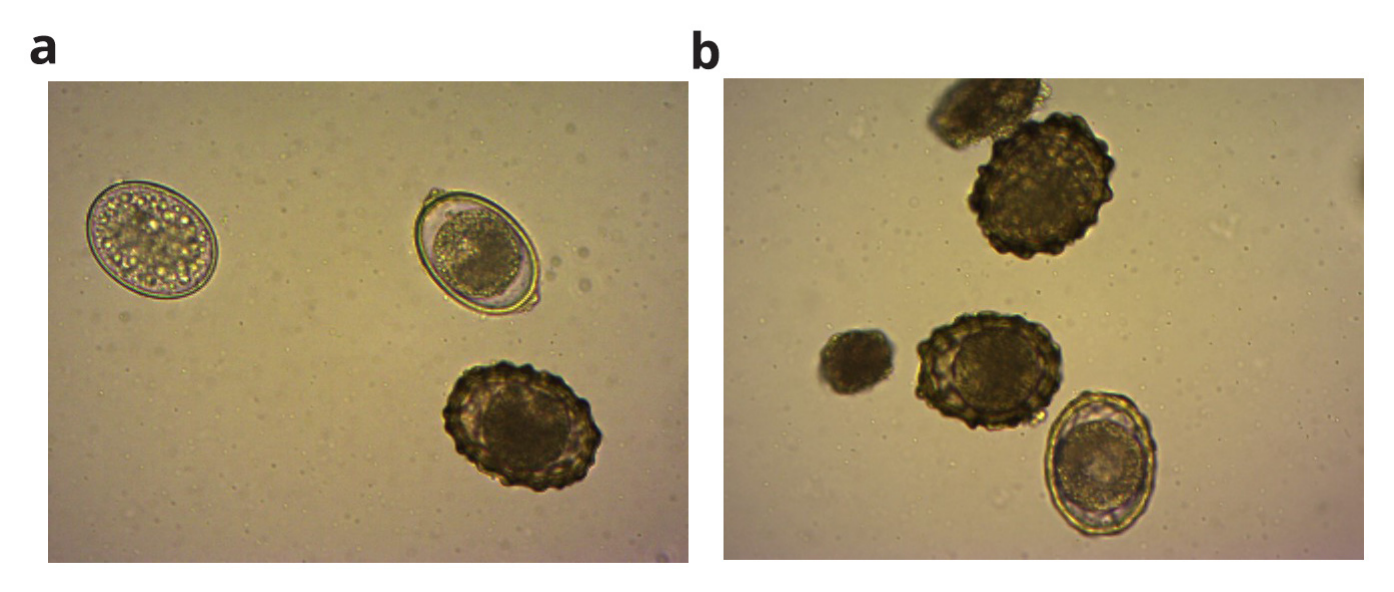
( **a**) and ( **b**): The viable and non-viable mixture of procured *Ascaris suum* eggs.

***Experimental setup.*** The biochemical methane potential (BMP) assay was performed in serum bottles (130 mL) to co-digest faecal sludge (Obtained after solid-liquid separation of septic tank sewage from the sewage treatment plant at Baina, Goa) and food waste (Collected from one of the messes of BITS Goa). The ratio of food waste to sludge in the bottles was 5:1. Before seeding the serum container, the sludge's total solid (TS) and volatile solid (VS) contents were evaluated. Each bottle was subjected to a loading rate of biomass of 1.5kg VS/m ^3^; the amount of faecal sludge and food waste to be added to the bottles to achieve desired loading rate was calculated using the VS values. VS was first calculated in kg/m ^3^ for a 5:1 ratio and then converted to grams/100mL as the final bottle volume was 100mL. Macro and micronutrients, sodium bicarbonate, digested from a large-scale anaerobic reactor (Plug flow reactor of 60 m ^3^ capacity constructed at BITS Goa for the treatment of food waste to produce biogas) as inoculum were added, and the final volume was raised to 100 mL by adding distilled water. Figure 1 shows the BMP bottle set up of the lab scale experiment. We conducted examinations on the inoculum (digested), faecal sludge, and food waste to detect the presence of helminth eggs before the inoculation process. None of the samples exhibited any trace of eggs. Consequently, we proceeded with the introduction of *A. suum* eggs into the system through spiking. *A. suum* eggs were procured from Excelsior Sentinel, USA, a mixture of viable and non-viable eggs ( Figure 2). Eggs of concentration 8 eggs/mL with approximately 63% viability were spiked in the BMP bottles. The experiment was conducted using 2 sets of bottles, each set contained 11 bottles named with the sampling day (Day 0, 3, 6, 9, 15, 20, 25, 30, 35, 40, and 45). One bottle from one set was taken for analysis. When the bottle was taken out at each sampling point, a gas reading from similar day set bottle was considered a duplicate reading to perform analysis. The setup was maintained at room temperature (28 ± 2°C).

***Parameter analysis.*** The different experimental parameters analyzed are given in Table 1. Biogas production was estimated daily by measuring the gas volume generated using a water displacement unit. A water displacement unit was made in the laboratory: a 5000 mL glass beaker was taken, and a 100 mL measuring cylinder was attached inside in an inverted position. The beaker was filled with distilled water until the zero mL mark on the cylinder, and gas from the bottle was passed inside the cylinder using a syringe with an IV tubing set. As the gas passed into the cylinder, it displaced the water, and the reading was recorded in mL. pH, TS, and VS analyses were conducted at each sampling point. pH was checked using an Oakton pH meter. The pH probe was dipped into the sample and the reading was taken. Readings were taken in duplicate. To plot a graph, standard deviation was calculated using the duplicate pH reading. For TS and VS analysis, the weight of the empty crucible was recorded, then the sample was added and the total weight was recorded. Sample weight was calculated by subtracting the crucible weight from the total weight (For the solid sample around 5 g of sample and for the liquid sample around 10 mL of sample was taken). Crucibles containing samples were kept at 105°C for 12 hours and the weight was recorded. Then the crucibles were ignited in a muffle furnace at 550°C for four hours and weighed once they had cooled down. TS was calculated by subtracting the crucible weight after the 105°C readings. VS was calculated by subtracting the 550°C weight values from the 105°C weights. Both TS and VS was then converted to per gram and then to percentage. The TS content was initially calculated and subsequently employed to determine the percentage of VS based on the TS value. To plot a graph, standard deviation was calculated using percentage values of both TS and VS.

**Table 1.** Parameters analysed during the biochemical methane potential (BMP) experiment.

	Parameters	Method/Instrument used
1.	Biogas	Water displacement unit
2.	pH	Oakton pH meter
3.	Total solids (TS)	Apha (2012)
4.	Volatile solids (VS)	Apha (2012)
5.	Volatile fatty acids (VFA)	Apha (1998)
6.	Biogas composition (CH _4_, CO _2_, H _2_S)	Gas chromatography (Trace 1110_Thermofisher Scientific)
7.	*A. suum* inactivation	Pebsworth *et al*. (2012)

Biogas composition (CH _4_, CO _2_, and H _2_S) was analyzed using gas chromatography (GC). The GC instrument (Trace 1110, Thermofisher Scientific) was equipped with a packed stainless-steel column of spherocarb support, a thermal conductivity detector (TCD), and hydrogen as the carrier gas. The injector temperature was set to 150°C and the detector temperature to 185°C. Gas sample was taken from BMP serum bottles using a 2 mL syringe and injected into the GC instrument. Gas composition was determined by following the steps in the software (Chrom-Card data system, Version 2.12, August 2014) present in the instrument itself. 

Volatile fatty acid, (VFA) analysis was conducted using the titration method ( Apha, 1998). A 5 mL sample was taken from the opened serum bottle, and the volume was raised to 100 mL using distilled water. The diluted sample was titrated in a glass beaker using 0.1 N HCl until the pH reached 3, and the burette reading (A) was recorded. Then 0.1 N NaOH was added until the pH reached 6.5, and the burette reading (B) was recorded. The following formula was used to calculate VFA (mg/L)


VFA(mg/L)={[(B∗101)−(A+100)]∗20∗60}/99.23(eq.1)

After taking the gas reading, TS and VFA analysis all the remaining sample was used for helminth analysis (75–85mL). For helminth eggs analysis, the sample was taken in a plastic beaker and ammonium bicarbonate solution was added; the sample was mixed on a magnetic stirrer (Cole-Parmer- Stuart-UC152D) for 20 min. The resultant solution was passed through a 100-micron sieve (Analysensieb test sieve, 200 mm diameter, Fritsch, Germany) kept on top of a 20-micron sieve. The sample was washed with tap water, and the contents of the 20-micron sieve were placed in four 15 mL centrifuge tubes and centrifugated at 3000 rpm in a swing out rotor centrifuge (Eltek, MP 800) for 10 min. After centrifugation, the supernatant was discarded and zinc sulphate solution of 1.3 specific gravity was added to the pellet to the 14 mL mark with 3 ml solution added at a time and mixed on a vortex (Neuation, Digital Vortex Mixer iSwix VT) and centrifuged again at 2000 rpm for 10 min. The supernatant was carefully placed in a small 20-micron sieve (100 mm diameter) and washed thoroughly. The contents of the sieve were transferred to a centrifuge tube, and centrifugated at 3000 rpm for 10 min. The pellet was made into suspension by adding distilled water (1–2 mL) for microscopy using distilled water (UKZN PRG helminth method ( Pebsworth *et al*., 2012). Using the ZEISS Primo Star microscope, eggs were observed and counted under 10X and 40X magnification. Images were captured by using iDS uEye Cockpit software (Version 4.96.1) which comes with the microscope. All parameters were examined in duplicate every third day for the first 15 days, then every fifth day until day 45. All the methods in detail are uploaded on Dryad ( Mutnuri & Patil, 2023).

***Statistical analysis.*** All the data were checked for normality and homogeneity of variance. The relationship between *A. suum* inactivation and all the other parameters was studied using correlation analysis. Statistical tests were performed using SPSS (IMB SPSS Statistics 25) software.

### Electrochemical treatment

**Figure 3.**
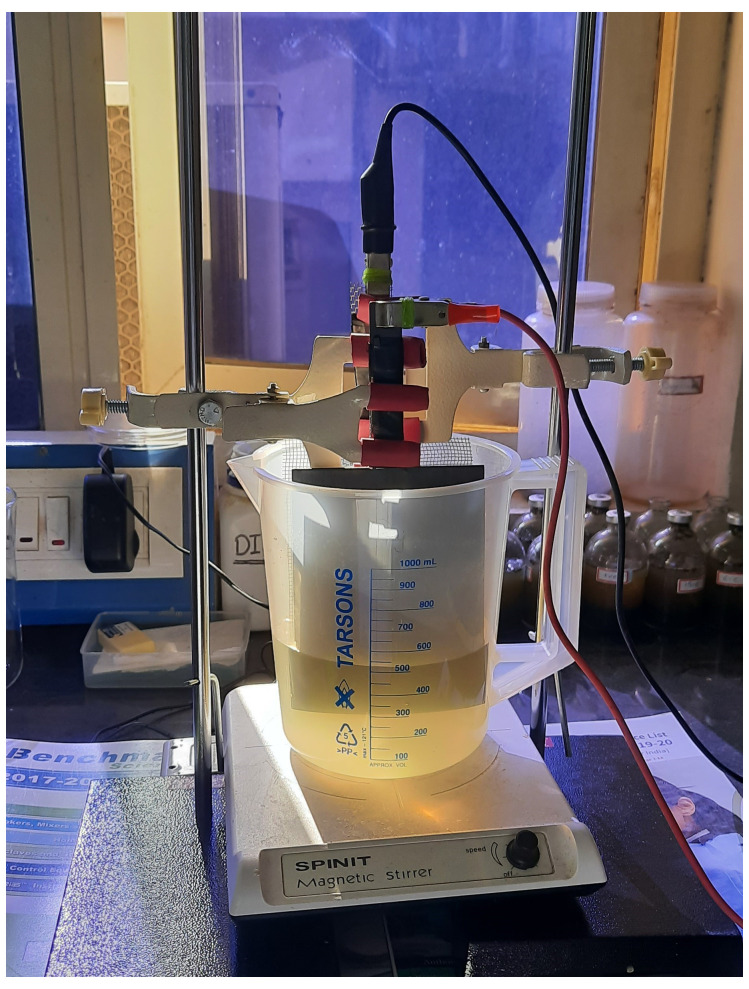
Electrochemical cell set up at lab scale.

***Experimental setup and experiment.*** The laboratory-scale electrochemical cell (EC) was set up in a 1000 mL beaker ( Figure 3). A titanium plate was used as an anode electrode, coated with 6 µm thick mixed metal oxide (Ruthenium, Iridium, and Titanium in 70%, 20%, and 10%, respectively). The cathode electrode was made of SS 304 stainless-steel mesh. A variable DC supply unit (Laboratory DC power supply, Gwinstek, GPS-4303) was used to apply a consistent voltage. The electrochemical cell solution was continuously mixed with a magnetic stirrer (SPINIT Motorless Magnetic Stirrer, Tarsons) to generate hypochlorite, which was then employed as a disinfectant for inactivating *A. suum* eggs.

Hypochlorite generation using NaCl has been done with different electrochemical processes ( Asokan & Subramanian, 2009; Spasojevic *et al*., 2015; Gogoi *et al*., 2022). This study generated hypochlorite as described in Gogoi *et al*., 2022 with a few modifications. Electrolysis of 2% NaCl solution was performed for two hours, producing 4700 ppm hypochlorite. The generated hypochlorite was diluted in various ratios with distilled water (1:5, 1:10, 1:20, 1:30, and hypochlorite without dilution). Helminth eggs (8 eggs/mL; 86% viability) were spiked into all diluted solutions, and helminth inactivation was observed under a microscope at 15 min, 30 min, hourly intervals between one and six hours, and 24 hours.

## Results

### Inactivation of *A. suum* eggs by anaerobic digestion

**Figure 4.**
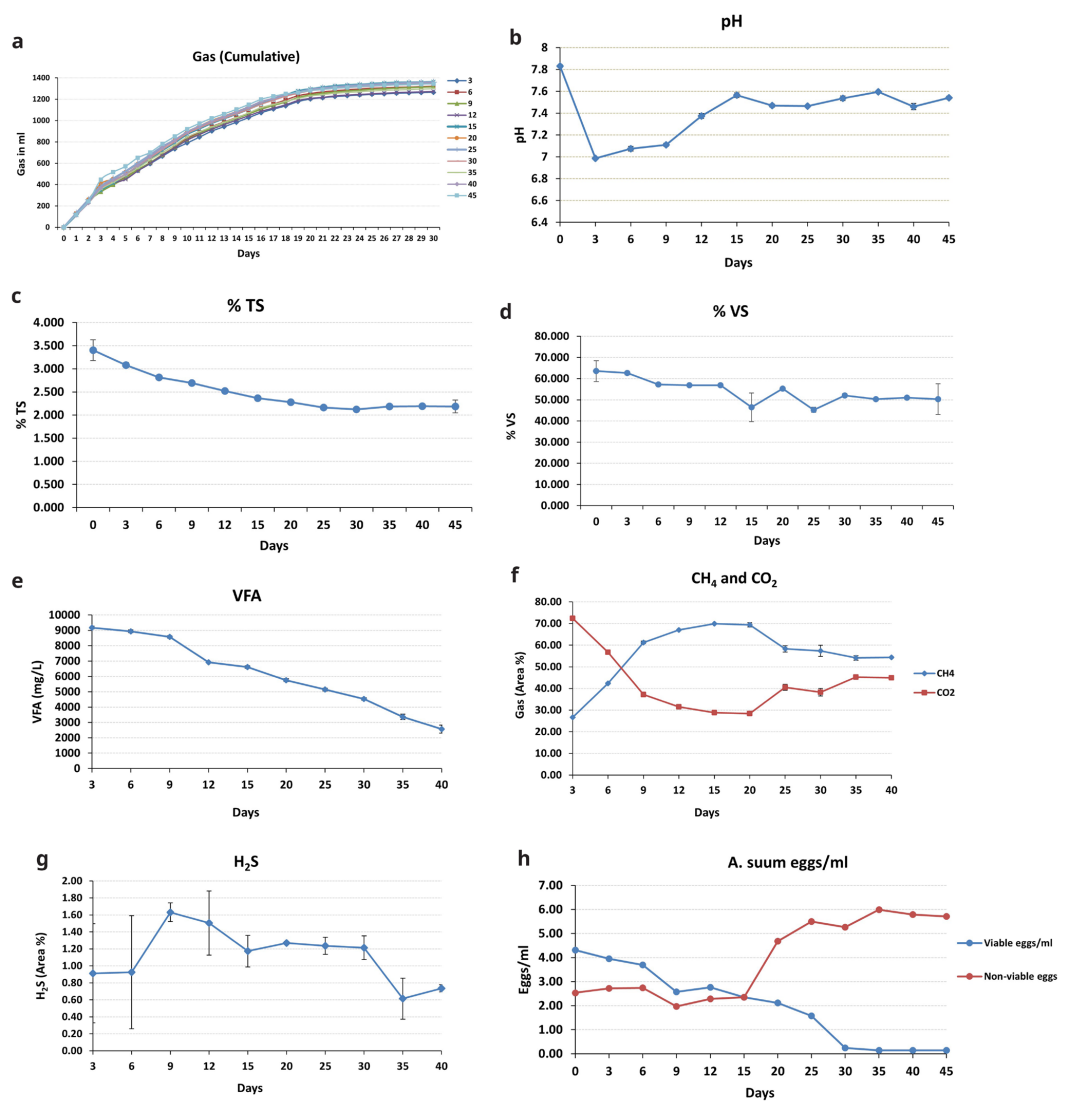
**a**) Cumulative biogas production, variations in the parameters **b**) pH, **c**) TS, **d**) VS, **e**) VFA, **f**) CH _4_, CO _2_, **g**) H _2_S, **h**) *A. suum* inactivation eggs/mL.

Figure 4 (a) depicts the biogas produced in the BMP assay. On the first day, the gas produced was between 112 and 122 mL, and on day 30, the overall gas generation (cumulative) was 1218 to 1389 mL. After 25 days, gas production began to decline; thus, recording readings were discontinued at 30 days. The produced gas was then examined using GC.

Throughout the experiment, the pH of the biomass remained approximately neutral (7–8) ( Figure 4(b)). It was 7.83 at the start of the experiment (day 0), but it dropped to 6.98 at the next sampling point (day 3). It then went up to 7.37 and remained between 7.3 and 7.5 for the remainder of the incubation period.

The amount of TS and VS in the biomass reduced as the incubation progressed ( Figure 4(c) and (d)). On day 0, TS was 3.4%; by day 45, it had dropped to 2.2%. VS followed a similar pattern, peaking at 63.5% on day 0 and dropping to 50.3% on the 45th day of incubation.

The VFA production in the biomass was highest on day 3 (9181 mg/L), which then decreased as the incubation period proceeded ( Figure 4 (e)), dropping down to 2572 mg/L, observed on the last sampling day.

The trends for CH _4_ and CO _2_ were diametrically opposed ( Figure 4 (f)). CH _4_ climbed to 69% until the 20th day, then declined to 54.3% until stabilizing on the 40 ^th^ day. CO _2_ levels were high at 72.4% at the first sampling point (day 3). It declined (28.4%) until the 20th day of incubation and then increased slightly until the experiment ended. Throughout the trial, H _2_S displayed an uneven trend ( Figure 4 (g)). The maximum H _2_S concentration (1.6%) was found on day 9, while the lowest (0.6%) was observed on day 35.

**Figure 5.**
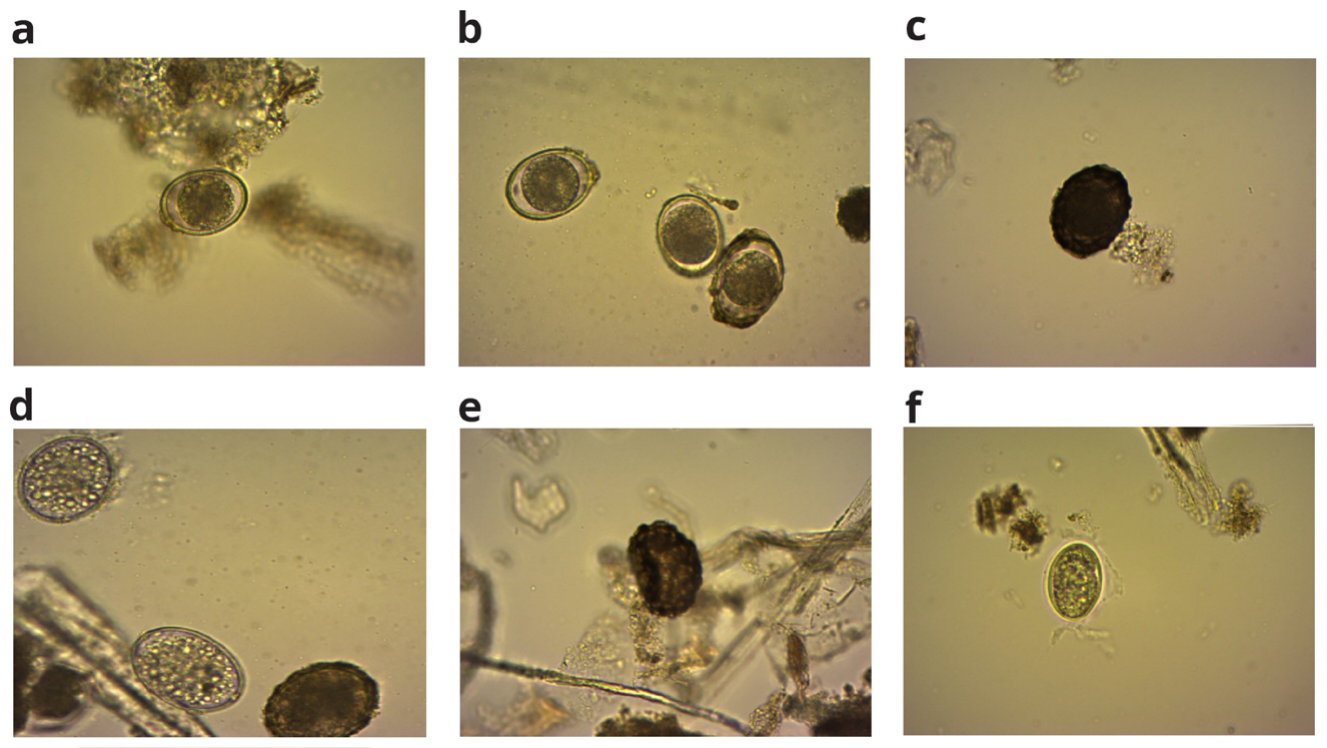
*A. suum* viable eggs ( **a**), ( **b**) and non-viable eggs ( **c**– **f**) on 45 ^th^ day of incubation.

The total number of viable *A. suum* eggs dropped over the course of the experiment, contributing to the increase in non-viable eggs ( Figure 4 (h)). The initial viable egg count was roughly 4 eggs/mL, reducing to 0.15 eggs/mL on the 35 ^th^ day (which meets the discharge standard according to WHO, 2006). Inactivation of eggs was relatively low for the first 15 days (2.35 eggs/mL) and then continued to increase, with 98% inactivation achieved by the 35th day of incubation. There was not much additional variation in the number of viable eggs after day 35 (0.14 eggs on the 40 ^th^ day); inactivation remained at 98%, so the experiment was stopped at 45 days. After 35 days, the number of non-viable eggs dropped marginally; this could be attributable to the complete disintegration of a few dead eggs that were not counted. Figures 5 (a–f) show representative pictures of *A. suum* on day 45.

### Effects of test parameters on *A. suum* eggs inactivation

Since the data for a few testing parameters was not normally distributed, Spearman's correlation analysis was used to individually analyze the relationship between the number of non-viable eggs and each test parameter. The analysis results demonstrated a moderate positive association between pH and non-viable eggs (rs = 0.465, p = 0.022), indicating that the number of non-viable cells will also increase as the pH rises. TS and VS had a substantial negative connection with non-viable eggs (rs = -0.752, -0.607, and p = 0.000, 0.002, respectively). VFA also had a strong negative correlation with non-viable eggs (rs = -0.840, p = 0.000), indicating that increasing VFA concentration alone will not contribute to the inactivation of helminth eggs. The CH _4_ and CO _2_ values were not statistically significant (p = 0.474, 0.563, respectively), indicating that the effect is insignificant in this case. H _2_S was negatively correlated with non-viable eggs (r = -0.555, p = 0.011).

### *A. suum* inactivation by the electrochemical (EC) process

**Figure 6.**
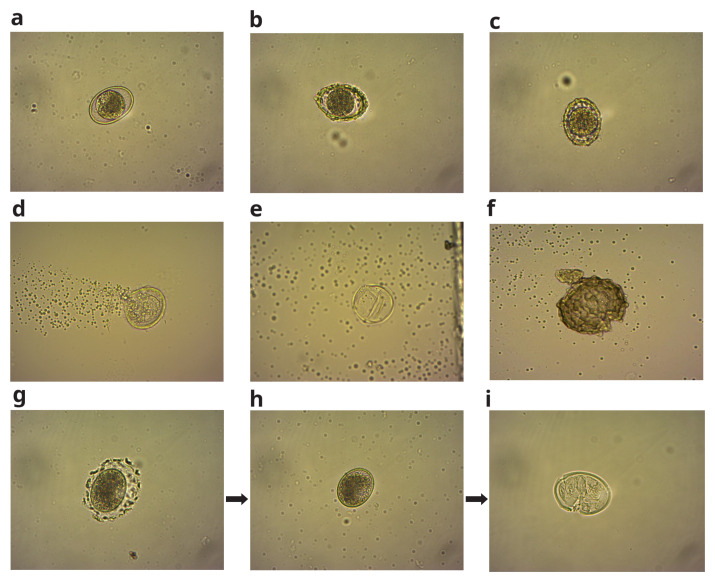
*A. suum* viable eggs ( **a**– **c**), and non-viable eggs ( **d**– **f**), from the EC experiment. ( **g**, **h** and **i**), show the process of dissolution of the outer part of the eggshell ( **g** and **h**) and subsequent membrane rupture ( **i**), resulting in complete inactivation of the egg in hypochlorite solution.

None of the different hypochlorite concentrations demonstrated evidence of helminth eggs being inactivated until six hours into incubation in the diluted hypochlorite solution. Only 10% inactivation was achieved in the 1:5 ratio (940 ppm) solution after a 24-hour incubation period. When exposed to absolute hypochlorite (without dilution), some of the *A. suum* eggs were quickly inactivated and over 24 hours, their inactivation was maximized. Table 2 shows the parameter values, and Figure 6 shows *A. suum* egg images before and after inactivation in hypochlorite solution.

**Table 2.** Variations in number of *A. suum* eggs during the EC experiment with time (Generated hypochlorite).

Ratio (Hypochlorite : Distilled water)	0 hr	15 min	30 min	1 h	2 h	3 h	4 h	5 h	6 h	24 h
	Number of *A. suum* eggs									
1:30 (156 ppm)	8	8	8	8	8	8	8	8	8	8
1:20 (235 ppm)	8	8	8	8	8	8	8	8	8	8
1:10 (470 ppm)	8	8	8	8	8	8	8	8	8	8
1:5 (940 ppm)	8	8	8	8	8	8	8	8	8	7.2
No dilution (4700 ppm)	8	7	7	6	5	5	4	3	3	2

## Discussion

The helminth inactivation study was conducted at a selected food waste and faecal sludge ratio. The ratio was selected as it demonstrated optimal biogas production in Shet and Mutnuri (2021). The BMP assay achieved 98% inactivation of *A. suum* eggs at the lab scale.

Pathogen inactivation in AD is as complex as the biogas generation process. Many parameters influence biogas production. In the current investigation, sodium bicarbonate was added to the serum bottles to limit the pH from becoming extremely acidic or basic (the pH stayed between 7–8), the extreme pH change might hinder the methane production. According to Pecson & Nelson (2005) and Fidjeland *et al*. (2015), In the absence of ammonia, basic pH (9–12) has no direct effect on the inactivation of *Ascaris* spp. Free ammonia (NH _3_) is considered more dangerous to pathogens because of its lipophilic nature, allowing it to penetrate through cell membranes without restriction, dissociate inside cells to shift ∆P across the cell membrane and generate an alkaline pH ( Pecson & Nelson, 2005). Spearman's correlation analysis revealed a positive correlation between pH and the number of non-viable eggs. This correlation results indicates, a basic pH is necessary for helminth inactivation, which aligns with what Mignotte-Cadiergues *et al*. (2001) observed. pH, in combination with high temperature, was also found to be effective in helminth egg inactivation ( Pecson *et al*., 2007). Maya *et al*. (2012) similarly noted comparable observations. Their findings suggested that the inactivation of helminth eggs can be achieved through the combined impact of pH, temperature, and dryness.

TS and VS do not directly impact helminth inactivation but are essential to track biogas generation. These parameters showed a strong negative correlation with non-viable *A.suum* count; their value decreased as the non-viable eggs increased.

Numerous studies have reported that VFA production helps in the inactivation of helminth eggs ( Puchajda *et al*., 2006; Rojas-Oropeza *et al*., 2017). It’s also reported that the free VFAs are more effective in helminth inactivation than ionized ones as they are lipophilic and membrane permeable ( Puchajda *et al*., 2006). The equilibrium of free and ionized VFA depends on the AD's pH and temperature ( Kunte *et al*., 2000). According to Riungu *et al*. (2018), the concentration of free VFA required to inactivate helminth eggs ranges from 4800 to 6000 mg/L. Despite reaching a certain concentration, VFA cannot inactivate eggs unless the pH is acidic ( Harroff *et al*., 2017). The maximum VFA concentration in this experiment achieved was 9181 mg/L, which is adequate to inactivate helminth eggs; perhaps because sodium bicarbonate was added to the serum bottle, the pH didn't change or turn acidic, and there was no positive effect shown in the data. Additionally, the biogas (CH _4_, CO _2_, and H _2_S) does not directly influence the inactivation of *A. suum*; this was validated by correlation analysis because the data were not statistically significant except for H _2_S.

*A. suum* inactivation in the electrochemical process was achieved at the highest (4700 mg/L) concentration of hypochlorite. Lower concentrations of hypochlorite were not efficient in deactivating HE, with only higher concentrations proving successful in deactivation (as depicted in Table 2). Nonetheless, these elevated concentrations lead to an increase in hypochlorite levels, rendering direct application impractical without additional treatment to reduce hypochlorite concentration. The results by Talekar *et al*., 2018 also indicated that chlorine concentration alone would not enhance inactivation. The inactivation of helminth eggs is influenced by a number of parameters, including the interaction of pH and free chlorine concentrations. Depending on the pH of the solution, hypochlorite persists in two forms (HOCl and OCl ^-^). The percentage of HOCl decreases, and the percentage of OCl ^-^ increases as the pH of the solution increases. HOCl is approximately eighty times more potent than OCl ^- ^because uncharged HOCl is more effective in penetrating cell walls. Additionally, compared to other chlorine-based disinfectants, it responds more quickly to oxidation processes involving the organic matter or the essential elements of microbial cells. In the current experiment, the highly concentrated hypochlorite was diluted in various ratios, producing a more basic final solution that reduced the inactivation rate of *A. suum* eggs.

Both treatment procedures (AD and EC process) are capable to inactivate HE. Drawing from the results of this study, we have compiled a list of advantages and disadvantages associated with both approaches as follows:

1. AD is a cost effective, easy to operate, sustainable treatment process which treats sludge and reduce greenhouse gas reduction making methane available for energy use. However, it has a long process time and it can be sensitive to changes in operating conditions.

2. EC process is an effective in pollutant removal, has a short operating time with potential for resource recovery. In spite of the short treatment efficiency, it has not been able to inactive HE with low hypochlorite concentration. The addition of chemicals can increase the overall treatment cost.

## Conclusions

In the present study, anaerobic digestion was successful in *A. suum* inactivation compared to the electrochemical process. Multiple factors influence inactivation. The following are the critical conclusion points from the present study.

1. Anaerobic digestion is helpful in the inactivation of helminth *(A. suum)* eggs.
2. The inactivation of *A. suum* eggs by anaerobic digestion is influenced by a combined effect of more than one factor.
3. Hypochlorite concentration or pH alone can’t deactivate helminth ( *A. suum*) eggs in the electrochemical treatment.
4. A detailed study with the high initial concentration of *A. suum* eggs can be helpful to achieve a complete inactivation.

## Data Availability

Dryad: Underlying data for ‘Study of helminth eggs ( *Ascaris suum*) inactivation by anaerobic digestion and electrochemical treatment’, https://doi.org/10.5061/dryad.rbnzs7hg4 ( Mutnuri & Patil, 2023) This project contains the following underlying data: A.suum_statistical_data__file_PP_and_SM_CSV.csv A._suum_PP_and_SM_CSV.csv Biogas_composition_(CH4__CO2_and_H2S)_PP_and_SM_CSV.csv Biogas_PP_and_SM_CSV.csv pH-PP_and_SM_CSV.csv Total_Solids_(TS)_and_Volatile_Solids_(VS)_PP_and_SM_CSV.csv Volatile_Fatty_Acids_(VFA)_PP_and_SM_CSV.csv README.md README.txt_File_PP_and_SM.txt Supporting_figures_PP_and_SM.pdf A._suum_stat_data_output_file_PP_and_SM.spv Supporting_tables_PP_and_SM.pdf Data are available under the terms of the Creative Commons Zero “No rights reserved” data waiver (CC0 1.0 Public domain dedication).

## References

[ref-1] APHA: Standards Methods for the Examination of Water and Wastewater. 20th edition, American Public Health Association, Washington, D.C.1998. Reference Source

[ref-2] Asokan K Subramanian K : Design of a tank electrolyser for in-situ generation of NaClO. In: *Proceedings of the World Congress on Engineering and Computer Science*. San Francisco, USA : WCECS.2009;1:139–142. Reference Source

[ref-3] Fidjeland J Nordin A Pecson BM : Modeling the inactivation of Ascaris eggs as a function of ammonia concentration and temperature. *Water Res.* 2015;83:153–160. 10.1016/j.watres.2015.06.030 26143272

[ref-4] Forés E Mejías-Molina C Ramos A : Evaluation of pathogen disinfection efficiency of electrochemical advanced oxidation to become a sustainable technology for water reuse. *Chemosphere.* 2023;313: 137393. 10.1016/j.chemosphere.2022.137393 36442679

[ref-5] Gogoi JK Sharma P Talekar GV : Effect of salt dosage on the performance efficiency of the electro-oxidation process employed for integrated blackwater treatment. *Int J Environ Sci Technol.* 2022;1–12. 10.1007/s13762-022-04528-7

[ref-6] Harroff LA Liotta JL Bowman DD : Inactivation of *Ascaris* eggs in human fecal material through in situ production of carboxylic acids. *Environ Sci Technol.* 2017;51(17):9729–9738. 10.1021/acs.est.7b02014 28759229

[ref-7] Jiménez B Maya C Sánchez E : Comparison of the quantity and quality of the microbiological content of sludge in countries with low and high content of pathogens. *Water Sci Technol.* 2002;46(10):17–24. 12479448

[ref-8] Kato S Fogarty E Bowman D : Effect of aerobic and anaerobic digestion on the viability of *Cryptosporidium parvum* oocysts and *Ascaris suum eggs*. *Int J Environ Health Res.* 2003;13(2):169–179. 10.1080/0960312031000098071 12745337

[ref-9] Kunte DP Yeole TY Ranade DR : Effect of volatile fatty acids on Shigella dysenteriae during anaerobic digestion of human night soil. *World J Microbiol Biotechnol.* 2000;16:519–522. 10.1023/A:1008915231175

[ref-10] Lin MH Mehraeen S Cheng G : Bacteria poration on modified boron-doped diamond electrode surfaces induced by divalent cation chelation. *Environ Sci Water Res Technol.* 2020;6(6):1576–1587. 10.1039/C9EW01108K

[ref-11] Machnicka A Grübel K : The effect of pre-treatment and anaerobic digestion for pathogens reduction in agricultural utilization of sewage sludge. *Environ Sci Pollut Res Int.* 2022;30(5):13801–13810. 10.1007/s11356-022-23164-9 36149557 PMC9898345

[ref-12] Manser ND Wald I Ergas SJ : Assessing the fate of *Ascaris suum* ova during mesophilic anaerobic digestion. *Environ Sci Technol.* 2015;49(5):3128–3135. 10.1021/es505807a 25679819

[ref-40] Maya C Torner-Morales FJ Lucario ES : Viability of six species of larval and non-larval helminth eggs for different conditions of temperature, pH and dryness. *Water Res.* 2012;46(15):4770–4782. 10.1016/j.watres.2012.06.014 22794801

[ref-13] Mignotte-Cadiergues B Maul A Huyard A : The effect of liming on the microbiological quality of urban sludge. *Water Sci Technol.* 2001;43(12):195–200. 10.2166/wst.2001.0737 11464755

[ref-14] Mutnuri S Patil P : Data from: Study of helminth eggs (Ascaris suum) inactivation by anaerobic digestion and electrochemical treatment. Dryad, [Dataset],2023. 10.5061/dryad.rbnzs7hg4 PMC11422576 39324031

[ref-15] Olsen JE Nansen P : Inactivation of some parasites by anaerobic digestion of cattle slurry. *Biological Wastes.* 1987;22(2):107–114. 10.1016/0269-7483(87)90043-7

[ref-16] Pebsworth PA Archer CE Appleton CC : Parasite transmission risk from geophagic and foraging behavior in chacma baboons. *Am J Primatol.* 2012;74(10):940–947. 10.1002/ajp.22046 22707091

[ref-18] Pecson BM Barrios JA Jiménez BE : The effects of temperature, pH, and ammonia concentration on the inactivation of *Ascaris* eggs in sewage sludge. *Water Res.* 2007;41(13):2893–2902. 10.1016/j.watres.2007.03.040 17524448

[ref-17] Pecson BM Nelson KL : Inactivation of *Ascaris suum* eggs by ammonia. *Environ Sci Technol.* 2005;39(20):7909–7914. 10.1021/es050659a 16295855

[ref-19] Popat SC Yates MV Deshusses MA : Kinetics of inactivation of indicator pathogens during thermophilic anaerobic digestion. *Water Res.* 2010;44(20):5965–5972. 10.1016/j.watres.2010.07.045 20692678

[ref-20] Puchajda B Oleszkiewicz J Sparling R : Low-Temperature Inactivation of Fecal Coliforms in Sludge Digestion. *Water Environ Res.* 2006;78(7):680–685. 10.2175/106143006x101638 16929637

[ref-21] Rice EW Bridgewater L American Public Health Association : Standard methods for the examination of water and wastewater. Washington, DC: American public health association.2012;10.

[ref-22] Riungu J Ronteltap M van Lier JB : Build-up and impact of volatile fatty acids on *E. coli* and *A. lumbricoides* during co-digestion of urine diverting dehydrating toilet (UDDT-F) faeces. *J Environ Manage.* 2018;215:22–31. 10.1016/j.jenvman.2018.02.076 29550544

[ref-23] Rojas-Oropeza M Hernández-Uresti AS Ortega-Charleston LS : Effect of volatile fatty acids in anaerobic conditions on viability of helminth ova ( *Ascaris suum*) in sanitization of municipal sludge. *Environ Technol.* 2017;38(17):2202–2208. 10.1080/09593330.2016.1254281 27784197

[ref-24] Ruiz-Espinoza JE Méndez-Contreras JM Alvarado-Lassman A : Effect of low temperature thermal pre-treatment on the solubilization of organic matter, pathogen inactivation and mesophilic anaerobic digestion of poultry sludge. *J Environ Sci Health A Tox Hazard Subst Environ Eng.* 2012;47(12):1795–1802. 10.1080/10934529.2012.689237 22755526

[ref-100] Salam N Azam S : Prevalence and distribution of soil-transmitted helminth infections in India. *BMC Public Health.* 2017;17(1):1–12. 10.1186/s12889-017-4113-2 28209148 PMC5311856

[ref-25] Shet R Mutnuri S : Anaerobic co-digestion of organic fraction of municipal solid waste and septage for sustainable waste treatment: a case study from Goa India. *Energy Res Soc Sci.* 2021;1(3):1–8. 10.24018/ejenergy.2021.1.3.15

[ref-26] Spasojević M Krstajić N Spasojević P : Modelling current efficiency in an electrochemical hypochlorite reactor. *Chem Eng Res Des.* 2015;93:591–601. 10.1016/j.cherd.2014.07.025

[ref-27] Talekar GV Sharma P Yadav A : Sanitation of blackwater via sequential wetland and electrochemical treatment. *npj Clean Water.* 2018;1(1): 14. 10.1038/s41545-018-0014-x

[ref-28] World Health Organization: WHO guidelines for the safe use of wasterwater excreta and greywater. World Health Organization,2006;1. Reference Source

[ref-29] World Health Organization: Research priorities for helminth infections: technical report of the TDR disease reference group on helminth infections. World Health Organization,2012. Reference Source 23420950

[ref-30] World Health Organization: Investing to overcome the global impact of neglected tropical diseases: third WHO report on neglected tropical diseases 2015. World Health Organization,2015;3. Reference Source

