# Supplementary material for: Study of helminth eggs ( Ascaris suum) inactivation by anaerobic digestion and electrochemical treatment
Source: Gates Open Res. 2024 Feb 12;7:93. Originally published 2023 Jun 12. [Version 2] doi: 10.12688/gatesopenres.14573.2 (PMC11422576; doi:10.12688/gatesopenres.14573.2)
Supplement: Peer review history

# Reviewer report: Reviewer response for version 2

**DOI:** 10.21956/gatesopenres.16588.r37941

**Published:** 2024-09-24

**Authors:** Isaac Dennis Amoah

**Affiliations:** 1 Durban University of Technology, Durban, South Africa; 2 University of Arizona, Tucson, USA

General Comments:

 1. Consistency in Italics for Scientific Names: Ensure that the species names like *Ascaris suum* are consistently italicized throughout the manuscript.

 2. Clarity in Study Aim: The introduction lacks clarity on whether the primary goal is to inactivate helminth eggs or to focus on biogas production. In various sections, the generation of biogas is emphasized, but without a clear explanation for its prioritization in a study primarily aiming to inactivate helminth eggs.

 3. Hypothesis Justification: The manuscript lacks a strong hypothesis regarding the role of anaerobic digestion (AD) in helminth egg inactivation. A stronger theoretical foundation should be established on why AD is expected to be effective for this purpose.

 4. Electrochemical Process (EC) and Controls: There is insufficient information on control samples during the electrochemical experiments. Without controls, it is difficult to determine the significance of the 10% inactivation rate reported. Consider adding a control group to demonstrate the baseline inactivation rate for comparison.

 Section-Specific Comments:

 Abstract:

 - Comment: The sentence "remained at 0.14 eggs/mL until day 45" lacks clarity. Are you referring to the total number of eggs or only viable eggs? It should be made clear that while the number of viable eggs might decrease, the total number (viable and inactivated) may remain constant.

 Introduction:

 - Comment: The sentence "Anaerobic digestion (AD) is one of the most comprehensive technologies used for the treatment of helminth eggs" is misleading. Anaerobic digestion is primarily used for wastewater treatment, not necessarily for helminth egg inactivation. This statement should be revised for accuracy.

 - Comment: Justification for the co-digestion with food waste is needed. The manuscript mentions biogas production as a concern, but if helminth egg inactivation is the primary focus, this needs further elaboration.

 - Comment: Reference missing for the statement on the prevalence of *Ascaris lumbricoides* in India.

 Methods:

 - Comment: It is important to specify the exact pH measurements taken during the experiment and clarify the significance of the pH change (neutral, acidic, or basic). Statistical tests applied to determine the significance of these changes should be mentioned explicitly.

 - Comment: Specify the voltage used during the electrochemical process as it has not been mentioned in the current description.

 Results:

 - Comment: It is unclear whether the results of the electrochemical treatment with hypochlorite achieved complete inactivation. A specific percentage should be given to define what "maximized" means in this context.

 - Comment: The correlation analysis section could be misleading. A weak positive correlation between pH and non-viable eggs is presented as significant, but this should be revisited for accuracy, and alternative statistical methods (like regression analysis) might yield more valuable insights.

 Discussion:

 - Comment: The interpretation of volatile fatty acids (VFA) inactivation is unclear. The negative correlation between VFA and non-viable eggs implies that VFA concentration does not directly contribute to inactivation. Clarify this interpretation.

 - Comment: The conclusions drawn from the electrochemical treatment are insufficient. Further elaboration on why hypochlorite concentration alone cannot deactivate helminth eggs is necessary. Mechanistic details of how electrochemical treatments might contribute to egg inactivation are expected.

 Conclusion:

 - Comment: The conclusion currently reads more like a summary of future directions. The findings should be more clearly defined, and the text should not focus heavily on future recommendations.

 Table 1:

 - Comment: APHA should be cited correctly and in uppercase. Additionally, the methodology used to calculate each parameter should be mentioned explicitly.

 Figures:

 - Comment: Figures 5 and 6 do not clearly show the impact of electrochemical treatment. Additional detail or a higher resolution image might help clarify the progression of egg inactivation.

 References:

 - Comment: The references are not arranged chronologically in some places. Ensure that they follow the correct order as required by the journal.

 Specific Statistical Corrections:

 1. Decimal Places: Where percentage values are presented, restrict the decimal places to one digit instead of two to ensure consistency and readability.

 2. Decimal Representation of Helminth Eggs: The use of decimals for egg counts should be reconsidered, as egg counts are whole numbers. Explain if decimal representation is necessary due to averaging over time or based on microscopy resolution.

 Further Recommendations:

 - Alkalinity: Alkalinity is a key stabilizing factor in anaerobic digestion. Although it was not measured in this study, future experiments should incorporate it to provide a more comprehensive analysis.

 - Mechanism of Inactivation: Provide further details on the mechanisms by which pH, VFAs, and other factors contribute to the inactivation of helminth eggs. This will help strengthen the scientific impact of the study.

 By addressing these comments, the manuscript will improve in clarity, scientific accuracy, and readability, thus enhancing its overall quality for indexing.

Is the work clearly and accurately presented and does it cite the current literature?

Partly

If applicable, is the statistical analysis and its interpretation appropriate?

Partly

Are all the source data underlying the results available to ensure full reproducibility?

Yes

Is the study design appropriate and is the work technically sound?

Partly

Are the conclusions drawn adequately supported by the results?

Partly

Are sufficient details of methods and analysis provided to allow replication by others?

Yes

Reviewer Expertise:

Environmental Microbiology, Water, Sanitation and Health (WASH), Health risk assessment, Antimicrobial resistance

I confirm that I have read this submission and believe that I have an appropriate level of expertise to confirm that it is of an acceptable scientific standard, however I have significant reservations, as outlined above.

# Reviewer report: Reviewer response for version 2

**DOI:** 10.21956/gatesopenres.16588.r35922

**Published:** 2024-02-27

**Authors:** Wanderli Rogério Moreira Leite

**Affiliations:** 1 Federal University of Pernambuco, Recife, State of Pernambuco, Brazil

The manuscript was revised by the authors and corrections were provided.

Is the work clearly and accurately presented and does it cite the current literature?

Yes

If applicable, is the statistical analysis and its interpretation appropriate?

Partly

Are all the source data underlying the results available to ensure full reproducibility?

Partly

Is the study design appropriate and is the work technically sound?

Yes

Are the conclusions drawn adequately supported by the results?

Yes

Are sufficient details of methods and analysis provided to allow replication by others?

Partly

Reviewer Expertise:

Anaerobic digestion, waste activated sludge, power-to-gas

I confirm that I have read this submission and believe that I have an appropriate level of expertise to confirm that it is of an acceptable scientific standard.

# Reviewer report: Reviewer response for version 1

**DOI:** 10.21956/gatesopenres.15889.r34171

**Published:** 2023-08-24

**Authors:** Yogalakshmi K.N.

**Affiliations:** 1 Central University of Punjab, Punjab, India

The manuscript is well written, but at some places the authors have missed out scientific language. The authors should do a complete spell check of the manuscript

 The specific comments on the manuscript include

1. At some places, the scientific name is not provided in italics
2. The references cited in the text are not arranged chronologically. 
3. What is the relevance of the following lines in the place provided (page 3, 4th para)
*Due to insufficient biogas production from the anaerobic digestion of faecal sludge alone, co-digestion of faecal sludge with food waste was carried out.*
4. pH is incorrectly written at certain places
5. Table 1 - APHA is not cited correctly.
6. The authors have provided emphasis on procedures that requires minimal explanation like pH, VFA, TS, VS etc. These are standard protocols and citing just the method is sufficient. The authors are advised to modify it. 
7. Give the photograph of BMP set up too.
8. Avoid two digits after decimal points in percentage. Restrict it to one digit.
9. Why didn't the authors measure alkalinity? Alkalinity is one of the important stabilizing parameter of AD process. Without alkalinity, interpreting pH and VFA and their impact on helminthes eggs will not be appropriate 
10. The results of TS and VS are wrong. How VS can be more than TS?
11. How can no. of helminthes eggs be given in decimals?
12. Why gas concentrations are given as area%? It is confusing.
13. Why is H2S gas monitoring given more importance?
14. pH and non viable eggs are showing weak positive correlation, how did the authors come to a conclusion with this value.
15. The interpretation of correlation analysis is misleading at many places. The authors should revisit the same.
16. The authors have claimed that adding sodium bicarbonate would inhibit methane, but the graph is showing good methane generation. The results and explanation are contradictory. 
17. Electrochemical studies should be elaborated more.
18. Conclusions should be be rewritten as it looks more like scope for further studies.
19. The mechanism behind the parameters influencing the non viability of helminth eggs should be provided. 

Is the work clearly and accurately presented and does it cite the current literature?

Yes

If applicable, is the statistical analysis and its interpretation appropriate?

Partly

Are all the source data underlying the results available to ensure full reproducibility?

No

Is the study design appropriate and is the work technically sound?

Yes

Are the conclusions drawn adequately supported by the results?

No

Are sufficient details of methods and analysis provided to allow replication by others?

Yes

Reviewer Expertise:

Water and waste water management, Circular economy, Solid waste management, Nanomaterials for Environmental applications

I confirm that I have read this submission and believe that I have an appropriate level of expertise to confirm that it is of an acceptable scientific standard, however I have significant reservations, as outlined above.

# Response

**Published:** 2023-12-30

**Authors:** Srikanth Mutnuri

**Affiliations:** Birla Institute of Technology and Science (BITS) K K Birla Goa Campus, Goa, India

Many thanks for your review. We've taken your suggestions into account and addressed each of your comments as follows: 

 1. At some places, the scientific name is not provided in italics

 Reply: Thank you pointing it out, we have corrected the same.

 2. The references cited in the text are not arranged chronologically.

 Reply: Thank you for the comment, wherever there are multiple references we have arranged them chronologically.

 3. What is the relevance of the following lines in the place provided (page 3, 4th para)

 Due to insufficient biogas production from the anaerobic digestion of faecal sludge alone, co-digestion of faecal sludge with food waste was carried out.

 Reply: The clarification - This research conducted a process of co-digestion, combining faecal sludge with food waste, aiming to enhance the biogas production. This approach was adopted due to concerns that using only faecal sludge might not generate an adequate amount of biogas. Clarification has been added in the introduction section.

 4. pH is incorrectly written at certain places

 Reply: Thank you for the observation, we have corrected it.

 5. Table 1 - APHA is not cited correctly.

 Reply: We have changed the citation of APHA.

 6. The authors have provided emphasis on procedures that requires minimal explanation like pH, VFA, TS, VS etc. These are standard protocols and citing just the method is sufficient. The authors are advised to modify it.

 Reply: In the initial submission of this research article, we had referenced the methods in a manner consistent with your suggestions. However, the editorial team subsequently requested that we provide a more detailed description of the methods to enhance the reproducibility of the experiments.

 7. Give the photograph of BMP set up too.

 Reply: We have included in the manuscript and provided it to the editorial team to incorporate in the paper.

 8. Avoid two digits after decimal points in percentage. Restrict it to one digit.

 Reply: The point has been noted and corrections are done wherever applicable.

 9. Why didn't the authors measure alkalinity? Alkalinity is one of the important stabilizing parameter of AD process. Without alkalinity, interpreting pH and VFA and their impact on helminthes eggs will not be appropriate.

 Reply: Thank you for your insightful observation. While alkalinity is indeed a crucial parameter in assessing the stability of the AD process, Due to some technical difficulties at the time of experiment we could not monitor it. In future investigations, incorporating alkalinity measurements could certainly provide a more holistic view of the AD process and its role in helminth eggs inactivation.

 10. The results of TS and VS are wrong. How VS can be more than TS?

 Reply: Sorry for the confusion induced by the representation, the percentage of VS is showing higher than TS in the results because the presented VS percentage pertains to the TS rather than the sample weight. The TS content was initially calculated and subsequently employed to determine the percentage of volatile solids based on the TS value. We have now included the specific information in the methods.

 11. How can no. of helminthes eggs be given in decimals?

 Reply: Initially, the eggs were enumerated as whole numbers during microscopy; however, as the experiment progressed and subsequent to the inactivation process, the low number of eggs made it unfeasible to accurately express the egg count per milliliter and the reduction percentage without employing decimal representation.

 12. Why gas concentrations are given as area%? It is confusing.

 Reply: The gas chromatography instrument employed in our experiment generated values in area percentages, which we have represented without any modifications. These values reflect the proportion of gases present as a percentage of the total. For instance, on day 6, the concentrations of CH _4_, CO _2_, and H _2_S were 61.3%, 37.1%, and 1.6% respectively, collectively summing up to 100%. This method of representation illustrates the relative composition of these gases in the overall sample, with each percentage denoting its portion in relation to the whole.   

 13. Why is H2S gas monitoring given more importance?

 Reply: If the comment is specific to the representation then we would like to clarify that, the graph for H _2_S was given separately as the gas percentage was very low compared to the CH _4_ and CO _2_, it was not possible to represent all three in one graph. According to the correlation analysis, the results were different that the CH _4_ and CO _2_. This distinction in representation was not intended to emphasize the importance of H _2_S but aimed at providing an accurate depiction of the findings.

 14. pH and non-viable eggs are showing weak positive correlation, how did the authors come to a conclusion with this value.

 Reply: The correlation can be regarded as moderate than weak as it falls in the range of “r 0.4-0.59”. While the correlation was identified as moderate is was considered because the data was statistically significant, it indicates a tendency for some level of relationship between these variables.

 15. The interpretation of correlation analysis is misleading at many places. The authors should revisit the same.

 Reply: Thank you for observation, we have now made changes in correlation analysis interpretation.

 16. The authors have claimed that adding sodium bicarbonate would inhibit methane, but the graph is showing good methane generation. The results and explanation are contradictory.

 Reply: Thank you for your observation. Our use of sodium bicarbonate aimed to stabilize pH within the optimal range (7-8) to prevent extreme pH fluctuations that could hinder methane production.

 In our experiment, addition of sodium bicarbonate effectively maintained the pH within a relatively stable range of 7 to 8, and that is why it showed good methane production. We acknowledge that our statement may have caused confusion; the intended message was to highlight the potential hindrance rather than complete inhibition of methane production in the case of extreme pH changes.

 17. Electrochemical studies should be elaborated more.

 Reply: Thank you for your suggestion. The conducted experiment did not demonstrate a positive potential for electrochemical treatment in helminth egg (HE) inactivation, which led to the decision to halt further experiments in this specific area. However, we acknowledge the importance of expanding on electrochemical studies, and we'll consider including this aspect in the future scope of our research. Exploring this avenue in subsequent studies could provide additional insights into the effectiveness of electrochemical methods for HE inactivation.

 18. Conclusions should be rewritten as it looks more like scope for further studies.

 Reply: Thank you for your comment, we have now made changes in the conclusion.

 19. The mechanism behind the parameters influencing the non-viability of helminth eggs should be provided.

 Reply: We acknowledge the significance of a detailed investigation into these factors. While our current study have shown the potential of these treatment methods, we recognize its relevance and inclusion for a comprehensive understanding. Investigating the intricate mechanisms governing the non-viability of helminth eggs is an integral part of our future research scope.

# Reviewer report: Reviewer response for version 1

**DOI:** 10.21956/gatesopenres.15889.r34165

**Published:** 2023-08-24

**Authors:** Wanderli Rogério Moreira Leite

**Affiliations:** 1 Federal University of Pernambuco, Recife, State of Pernambuco, Brazil

The manuscript present results of a research on helminth eggs (HE) inactivation using different approaches. The main outcomes were described, and some statistical analyses were used to correlate AD features to the effectiveness of HE inactivation. I would recommend that the article is copy-edited. I consider the manuscript acceptable for indexing after revision. Here are some major revisions:

- There is no citation to the VFA analysis method.
- Authors must enhance the discussion over the correlation between TS, VS and pH, H2S in biogas on A. suum eggs inactivation. Moreover, statistical analysis to link the behavior of the electrochemical reactor and A. suum eggs inactivation are missing.
- For what do numbers in Table 2 stand? Caption must be improved, and the discussion should include refer to this table.
- Arrows in Figure 5 give the idea of a process depicted in pictures. Authors must clarify the idea, either remove or justify arrows in the Figure.
- Authors performed HE inactivation using anaerobic digestion and electrochemical procedures, but did not compare the results, advantages and disadvantages of both approaches.
- Helminth eggs are incubated at 28°C–30°C for up to 28 days depending on the helminth, to allow the viable ova to hatch. Did authors consider this approach to state about viable HE?
- Recent research has depicted Acylostoma eggs were predominant in a sewage sludge digestate. The unexpected fact was addressed to some reactor operating parameters (Leite et al. 2023 Mesophilic anaerobic digestion of waste activated sludge […]. J Env Managem [ref-1]). The authors considered A. suum eggs as the main helminth egg found in fecal sludge. All materials and methods for their detection and inactivation were then described. However, the article I am proposing presents results that instead of Ascaris eggs, Acylostoma eggs were predominant in sewage sludge after anaerobic digestion. In my opinion, the authors should disclose in the introduction section that the occurrence of HE in a digestate is related to either health standards or operating conditions applied to the anaerobic digestion system.
  Authors are encouraged to consider this fact and include the information of how much sample was used to perform the HE analysis. Moreover, did the inoculum or the substrates were checked regarding the HE characterization? 

Is the work clearly and accurately presented and does it cite the current literature?

Yes

If applicable, is the statistical analysis and its interpretation appropriate?

Partly

Are all the source data underlying the results available to ensure full reproducibility?

Partly

Is the study design appropriate and is the work technically sound?

Yes

Are the conclusions drawn adequately supported by the results?

Yes

Are sufficient details of methods and analysis provided to allow replication by others?

Partly

Reviewer Expertise:

Anaerobic digestion, waste activated sludge, power-to-gas

I confirm that I have read this submission and believe that I have an appropriate level of expertise to confirm that it is of an acceptable scientific standard, however I have significant reservations, as outlined above.

# Response

**Published:** 2023-12-30

**Authors:** Srikanth Mutnuri

**Affiliations:** Birla Institute of Technology and Science (BITS) K K Birla Goa Campus, Goa, India

Many thanks for your feedback. We've implemented your suggestions and addressed each of your comments as outlined below:

 1. There is no citation to the VFA analysis method

 Reply: Reference to the VFA method was provided in the table 1, we have also added it in the text.

 2. Authors must enhance the discussion over the correlation between TS, VS and pH, H _2_S in biogas on *A. suum* eggs inactivation. Moreover, statistical analysis to link the behavior of the electrochemical reactor and *A. suum* eggs inactivation are missing.

 Reply: Thank you for your valuable comment. We have taken note and included additional discussion points concerning the correlation between TS, VS, pH, H _2_S in biogas, and their impact on *A. suum* egg inactivation. Regarding the statistical analysis concerning the behavior of the electrochemical reactor and *A. suum* egg inactivation, we acknowledge the importance of statistical analysis in this scientific investigation. However, the number of helminth eggs indicated in the table exhibited minimal diversity (Inactivation was not achieved for the diluted hypochlorite concentrations resulting in showing the same number of eggs for most of the data points) among the different concentrations of hypochlorite and time intervals. This lack of substantial diversity led to inconclusive outcomes from the statistical tests, unable to offer significant insights beyond what has already been detailed in the reported outcomes.

 3. For what do numbers in Table 2 stand? Caption must be improved, and the discussion should include refer to this table.

 Reply: Thank you for the comment, we have made changes in the table 2, improved caption and we have included points in discussion section.

 4. Arrows in Figure 5 give the idea of a process depicted in pictures. Authors must clarify the idea, either remove or justify arrows in the Figure.

 Reply: Thank you for pointing it out, we have informed the editorial team to make the necessary changes in figure 5, also the description of the figure is modified.

 5. Authors performed HE inactivation using anaerobic digestion and electrochemical procedures, but did not compare the results, advantages and disadvantages of both approaches.

 Reply: When we carried out the study our aim was to use different methods for *A. suum* inactivation, we did not intend to compare the methods. However we have taken your suggestion and now added a brief comparison along with major advantages and disadvantages in the discussion section.

 6. Helminth eggs are incubated at 28°C–30°C for up to 28 days depending on the helminth, to allow the viable ova to hatch. Did authors consider this approach to state about viable HE?-

 Reply: The status of eggs (Viable or live, Non-viable or dead) was checked only microscopically soon after the extraction from the sample.

 7. Recent research has depicted Acylostoma eggs were predominant in a sewage sludge digestate. The unexpected fact was addressed to some reactor operating parameters (Leite et al. 2023 Mesophilic anaerobic digestion of waste activated sludge […]. J Env Managem [ref-1]). The authors considered A. suum eggs as the main helminth egg found in fecal sludge. All materials and methods for their detection and inactivation were then described. However, the article I am proposing presents results that instead of Ascaris eggs, Acylostoma eggs were predominant in sewage sludge after anaerobic digestion. In my opinion, the authors should disclose in the introduction section that the occurrence of HE in a digestate is related to either health standards or operating conditions applied to the anaerobic digestion system.

 Authors are encouraged to consider this fact and include the information of how much sample was used to perform the HE analysis. Moreover, did the inoculum or the substrates were checked regarding the HE characterization?

 Reply: Apologies for the misinterpretation. Our prior literature review identified *A. lumbricoides* as the predominant helminth species present in faecal sludge (as that is the most prevalent infection in India), not *A. suum*. However, given the resemblance and comparable response to treatment methods observed in the literature, we opted to use *A. suum* in this study as a surrogate for *A. lumbricoides *(Many studies have been conducted using *A. suum*). References for the same are given in the introduction section. The clarification is included in the introduction section.

 It is correct that the occurrence of HE in the digested is related to either health standard or operating conditions applied. In this study, we conducted examinations on the inoculum, faecal sludge, and food waste to detect the presence of helminth eggs before the inoculation process. None of the samples exhibited any trace of eggs. Consequently, we proceeded with the introduction of *A. suum* into the system through spiking.

 Suggested changes are made in the introduction and methods sections.
